# Comparative analysis of gut microbiota of mosquito communities in central Illinois

**DOI:** 10.1371/journal.pntd.0005377

**Published:** 2017-02-28

**Authors:** Ephantus J. Muturi, Jose L. Ramirez, Alejandro P. Rooney, Chang-Hyun Kim

**Affiliations:** 1 Crop Bioprotection Research Unit, Agricultural Research Service, U.S. Department of Agriculture, Peoria, IL United States of America; 2 Illinois Natural History Survey, University of Illinois at Urbana-Champaign, Champaign IL. United States of America; Mahidol University, THAILAND

## Abstract

**Background:**

The composition and structure of microbial communities that inhabit the mosquito midguts are poorly understood despite their well-documented potential to impede pathogen transmission.

**Methodology/Principal findings:**

We used MiSeq sequencing of the 16S rRNA gene to characterize the bacterial communities of field-collected populations of 12 mosquito species. After quality filtering and rarefaction, the remaining sequences were assigned to 181 operational taxonomic units (OTUs). Approximately 58% of these OTUs occurred in at least two mosquito species but only three OTUs: *Gluconobacter* (OTU 1), *Propionibacterium* (OTU 9), and *Staphylococcus* (OTU 31) occurred in all 12 mosquito species. Individuals of different mosquito species shared similar gut microbiota and it was common for individuals of the same species from the same study site and collection date to harbor different gut microbiota. On average, the microbiota of *Aedes albopictus* was the least diverse and significantly less even compared to *Anopheles crucians*, *An*. *quadrimaculatus*, *Ae*. *triseriatus*, *Ae*. *vexans*, *Ae*. *japonicus*, *Culex restuans*, and *Culiseta inornata*. The microbial community of *Cx*. *pipiens* and *Ae*. *albopictus* differed significantly from all other mosquitoes species and was primarily driven by the dominance of *Wolbachia*.

**Conclusion and significance:**

These findings expand the range of mosquito species whose gut microbiota has been characterized and sets the foundation for further studies to determine the influence of these microbiota on vector susceptibility to pathogens.

## Author summary

The microbial communities that reside in mosquito midguts can impact transmission of mosquito-borne pathogens. We used high throughput next generation sequencing to characterize the midgut microbial communities of 12 mosquito species collected in urban residential areas in Champaign County, Illinois. A total of 181 OTUs from 11 phyla and 66 families were identified. Although several bacterial taxa were shared between two or more mosquito species, there was remarkable individual differences in gut microbiota and it was common for individuals of different mosquito species to harbor similar gut microbiota. The microbiota of *Ae*. *albopictus* was the least diverse and significantly less evenly distributed compared to 7 of 11 mosquito species. The microbial community of *Cx*. *pipiens* and *Ae*. *albopictus* differed significantly from other mosquito species and was primarily dominated by *Wolbachia*. These findings improve current knowledge on the composition and structure of mosquito gut microbiota and provide the framework for understanding their contribution to individual variation in vector competence and potential application in disease control.

## Introduction

Mosquitoes transmit a wide range of pathogens that cause diseases in humans and other animals. The majority of mosquito-borne pathogens were previously confined to small geographic regions in the tropics but have recently emerged as a worldwide threat to human and animal health. Recent examples of mosquito-borne diseases that have caused major epidemics outside their native geographic range include West Nile virus [1], dengue virus [2], Chikungunya virus [3] and Zika virus [4, 5].

The transmission cycle of mosquito-borne pathogens involve interactions between at least three species: the pathogen, the vector, and the vertebrate host. When the mosquito takes a blood meal from an infected vertebrate host, the pathogen invades the midgut tissue where it undergoes further development and/or replication and then disseminates to secondary tissues such as nerve tissue, fat body, and finally the salivary glands [6]. At this point, the mosquito is considered infectious and is capable of transmitting the pathogen during a subsequent blood meal. However, the mosquito midgut is known to possess factors that may impede successful transmission of the pathogen [7–10]. These factors include the mosquito innate immune system and the digestive enzymes [6, 8, 11].

It is also well established that the mosquito midgut is colonized by a community of bacteria that can affect vector susceptibility to pathogens e.g. [12, 13]. For example, certain bacterial isolates from natural mosquito populations have been shown to reduce mosquito susceptibility to *Plasmodium* and dengue infection [12, 14, 15]. These effects are exerted through activation of the mosquito immune system [16], generation of reactive oxygen species by certain microbes [15], and formation of a physical barrier to infection [17]. Likewise, modification of midgut microbiota of *Anopheles gambiae* and *Aedes aegypti* through antibiotic treatment has been shown to enhance susceptibility to *Plasmodium* [16] and dengue infection [18], respectively. Other studies have shown that some midgut bacterial isolates can be genetically modified to express molecules that impair pathogen development within the mosquito [19, 20]. Collectively, these findings suggest that the composition of mosquito midgut microbiota likely contributes to within- and between-species variation in vector competence that is typical of many (if not all) mosquito-borne disease systems. Moreover, these studies demonstrate the potential for exploiting microbial functions for symbiotic control of mosquito-borne diseases [21].

Over the last few decades numerous studies have used culture-dependent and culture-independent approaches to characterize the microbial communities in the midguts of mosquito populations. These studies have revealed that the composition and diversity of gut microbiota can vary dramatically within [22] and between mosquito species [23] and are influenced by host diet [24], developmental stage [24], larval environment [25], and pathogen infection [26, 27]. As such, additional studies comparing the microbial communities of different mosquito species can further improve our understanding of mosquito microbiota and propel identification of specific microbes that may be harnessed for disease control.

In this study, we characterized the microbiota of 12 mosquito species collected from Champaign County, Illinois. The aim of this study was to determine how gut microbial diversity, composition and structure differs between mosquito species. Overall, we observed some remarkable similarities in gut microbiota between individuals of different mosquito species that were dominated by one or two bacterial taxa. These bacterial communities tended to vary markedly between individuals. We also found significant differences in bacterial community structure between some mosquito species. These findings advance current knowledge on the microbial communities that reside in mosquito midguts and provide the foundation for investigating their role in mosquito biology and potential application in mosquito-borne disease control.

## Materials and methods

### Mosquito collection

**Fig 1:**
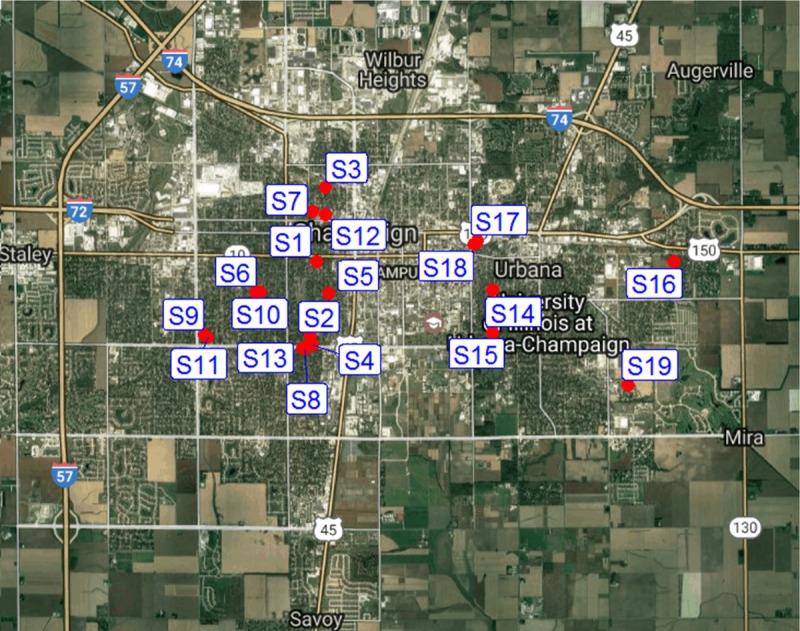
Map of the study area showing the location of the traps. S = trap site, S1 = 504 S Lynn St, Champaign IL; S2 = 1509 Grandview Dr, Champaign IL; S3 = 408 W Maple St, Champaign IL; S4 = 1809 Grandview Dr, Champaign IL; S5 = 805 S. Prairie St, Champaign IL; S6 = 1116 Charles St, Champaign IL; S7 = 604 W Hill St, Champaign IL; S8 = 1605 Coronado Dr, Champaign IL; S9 = 1413 S Western Ave, Champaign IL; S10 = 909 S McKinley, Champaign IL; S11 = 1418 S Western Ave, Champaign IL; S12 = 409 W Hill St, Champaign IL; S13 = 705 W Kirby Ave, Champaign IL; S14 = 602 Nevada St, Urbana IL; S15 = 602 Vermont Ave, Urbana IL; S16 = 2402 E Elm St, Urbana IL; S17 = 804 W Main St, Urbana IL; S18 = 807 W Main St, Urbana IL; S 19 = 2010 Morrow Ct, Urbana IL.

Mosquito samples for this study were collected once per week (July 2, 2015 to October 15, 2015) outside 19 urban residential houses in Champaign County, Illinois with permission from property owners (Fig 1). The sites were located within a 10 km radius of each other. The collections were done using standard CDC miniature light traps that were baited with dry ice as an attractant. The traps were tied to a tree outside the respective houses and operated between 1800 hours and 0900 hours. Mosquitoes from each trap were transported live in cool boxes, identified morphologically to species [28], and stored at -80°C until further processing.

### Midgut dissection, DNA extraction and 16S rRNA gene library preparation

Individual female mosquitoes were surface sterilized as previously described [23] and dissected in 50 μl of Dulbecco’s phosphate buffered saline (DPBS) solution (Thermo Fisher Scientific, Waltham, MA). Total DNA was isolated from each midgut using QIAamp DNA mini kit (Qiagen, Valencia, CA). A portion of DNA from *Culex* mosquitoes was used for species identification using real-time polymerase chain reaction [29]. In total, 264 midguts from 12 mosquito species were processed (Table 1). The V3-V5 region of the 16S rRNA gene was amplified and sequenced using Illumina MiSeq Bulk v3 platform at the W. M. Keck Center for Comparative and Functional Genomics at the University of Illinois at Urbana-Champaign as previously described [23]. The following primer set was used: forward 5ʹ -CCTACGGGAGGCAGCAG-3`and reverse 5`-CCGTCAATTCMTTTRAGT-3ʹ.

**Table 1:** Number of midgdut samples that were processed for each of the 12 mosquito species collected in Urbana-Champaign, IL.

	Number of midgut samples	
Mosquito species	Initial	Final
*Aedes albopictus*	28	27
*Aedes japonicus*	28	21
*Aedes triseriatus*	28	23
*Aedes trivittatus*	28	18
*Aedes vexans*	27	27
*Anopheles crucians*	27	15
*Anopheles punctipennis*	27	14
*Anopheles quadrimaculatus*	27	15
*Culex pipiens*	16	15
*Culex restuans*	8	4
*Culiseta inornata*	11	10
*Psorophora ferox*	9	6
	264	195

In brief, all DNA samples were measured on a Qubit (Life Technologies) using High Sensitivity DNA Kit and diluted to 2 ng/μl. A master mix containing 0.5 μl -10X FastStart Reaction Buffer without MgCl_2_, 0.9 μl -25 mM MgCl_2_, 0.25 μl -DMSO, 0.1 μl -10 mM PCR grade Nucleotide Mix, 0.05 μl -5 U/μl FastStart High Fidelity Enzyme Blend, 0.25 μl -20X Access Array Loading Reagent, and 0.95 μl -water was prepared using the Roche High Fidelity Fast Start Kit and 20X Access Array loading reagent and aliquoted into 48 well PCR plates along with 1 μl DNA sample and 1 μl Fluidigm Illumina linkers (V3-V5-F357: ACACTGACGACATGGTTCTACA and V3-V5-R926:TACGGTAGCAGAGACTTGGTCT) with unique barcode. In a separate plate, primer pairs were prepared and aliquoted. 20X primer solutions were prepared by adding 2 μl of each forward and reverse primer, 5 μl of 20X Access Array Loading Reagent and water to a final volume of 100 μl.

Four μl of sample was loaded in the sample inlets and 4 μl of primer loaded in primer inlets of a previously primed Fluidigm 48.48 Access Array IFC. The IFC was placed in an AX controller (Fluidigm Corp.) for microfluidic loading of all primer/sample combinations. Following the loading stage, the IFC plate was loaded on the Fluidigm Biomark HD PCR machine and samples were amplified using the following Access Array cycling program without imaging: 50°C for 2 minutes (1 cycle), 70°C for 20 minutes (1 cycle), 95°C for 10 minutes (1 cycle), followed by 10 cycles at 95°C for 15 seconds, 60°C for 30 seconds, and 72°C for 1 minute, 2 cycles at 95°C for 15 seconds, 80°C for 30 seconds, 60°C for 30 seconds, and 72°C for 1 minute, 8 cycles at 95°C for 15 seconds, 60°C for 30 seconds, and 72° for 1 minute, 2 cycles at 95°C for 15 seconds, 80°C for 30 seconds, 60°C for 30 seconds, and 72°C for 1 minute, 8 cycles at 95°C for 15 seconds, 60°C for 30 seconds, and 72°C for 1 minute, and 5 cycles at 95°C for 15 seconds, 80°C for 30 seconds, 60°C for 30 seconds, and 72°C for 1 minute. The PCR product was transferred to a new 96 well plate, quantified on a Qubit fluorimeter (Thermo-Fisher) and stored at -20°C. All samples were run on a Fragment Analyzer (Advanced Analytics, Ames, IA) and amplicon regions and expected sizes confirmed. Samples were then pooled in equal amounts according to product concentration. The pooled products were size selected on a 2% agarose E-gel (Life Technologies) and extracted from the isolated gel slice with QIAquick gel extraction kit (QIAGEN). Cleaned size selected products were run on an Agilent Bioanalyzer to confirm appropriate profile and determination of average size. The final library pool was spiked with 10% non-indexed PhiX control library (Illumina) and sequenced using Illumina MiSeq V3 Bulk system. The libraries were sequenced from both ends of the molecules to a total read length of 300nt from each end. Cluster density was 964k/mm2 with 85.9% of clusters passing filter.

### OTU picking and taxonomy assignment

IM-TORNADO 2.0.3.2 platform was used to process the de-multiplexed fasq-formatted files obtained from the sequencing facility. This platform is designed to process non-overlapping reads for analysis as a whole unit without sacrificing one of the reads in the pair and improves accuracy in read analysis compared to single-end read analysis [30]. The 5ʹ PCR primer for forward (R1) and reverse (R2) reads were trimmed using Trimmomatic program [31] with the parameter HEADCROP:17 for R1 read and HEADCROP: 18 for R2 read. The quality filtering process was performed using Trimmomatic program following previously described procedures with slight modifications [30]. Briefly, the sequences were subjected to a hard cutoff of PHRED score Q3 for 5 ʹ and 3ʹ ends of the reads (parameters LEADING: 3 and TRAILING: 3), trimming of the 3’ end with a moving average score of Q15, with a window size of four bases (parameter SLIDINGWINDOW: 4:15), and any reads with less than 150 base pairs removed with parameter R1_TRIM = 150 and R2_TRIM = 150. Reads with ambiguous base calls were discarded. To retain both reads while avoiding misinterpretation of the data, matching R1 and R2 reads were joined using an ambiguous nucleotide character “N” between R1 and R2 [30]. In a single run, IM-TORNADO generates outputs for R1 data only, R2 data only, and paired end data. Only output files related to paired end data were used for taxonomic assignment and downstream analysis. Reads were de-replicated building clusters of reads with 100% similarity and annotated with cluster size. Singletons and reads shorter than the cutoff length were discarded to ensure the use of high quality reads when assigning OTU representation. Reads were sorted by cluster size and processed in USEARCH using the UPARSE algorithm to find the OTU representatives using *de novo* OTU picking strategy. Chimeric reads are also removed during this step resulting in a set of OTU representatives of very high sequence quality [32]. Operational taxonomic units (OTUs) were assigned at 97% sequence similarity using the Ribosomal Database Project (RDP) version 10 as the reference set with a threshold of 80% bootstrap confidence [33].

### PCR validation for *Wolbachia* surface antigen, *wsp*

Quantitative TaqMan real-time PCR (qPCR) was used to confirm the *wsp* gene of *Wolbachia* in mosquito midgut samples using the following primer set: forward: 5’-GSTTTTGCTKRTCAAGYAARAG-3’ and reverse: 5’-GYGCTGTAAAGAACKTTGWDY-3' respectively. Taqman probe sequence was 5’ FAM-TGTTAGTTATGATGTAACTCCRGAA-IABFQ 3’. The primers and probe were synthesized by Integrated DNA Technology, Inc. (IDT, Coralville, IA). Twenty microliter qPCR contained 1× SensiFAST Probe Hi Rox mastermix (BioLine, Taunton, MA), 0.5 μM of each primer, 0.25 μM Taqman probe and 2 μL of the mosquito midgut DNA isolate. The qPCR was run with 1 cycle of heat activation at 95°C for 15 minutes followed by 45 cycles of denaturation at 94°C for 1 minute, annealing at 50°C for 1 minute and elongation at 72°C for 1 minute.

Minigene was constructed using *wsp* gene segment flanked by the PCR primers and was synthesized by IDT (Coralville, IA). The gene sequences utilized for the minigene were downloaded from GenBank and the accession number was CP001391 for *Wolbachia* spp wRi. The minigene was used as a positive control for qPCR of *Wolbachia wsp* gene and as templates for building a standard curve to estimate the quantity of *wsp* gene in mosquito midgut samples. The copy number of minigene (2063 bp) containing *wsp* gene segment was calculated based on the DNA concentration determined by NanoDrop 1000 spectrophotometer (Thermo Scientific) and on the assumption that the average weight of a DNA base pair (bp) is 650 Daltons. The formula for copy number calculation is: copy numbers = ((minigene amounts in ng) × (6.022 × 10^23^)) / (2063 × 650 × 10^9^). The concentration of minigene solution was adjusted to be 5 × 10^9^ copies/μl and 10-fold serially diluted in nuclease free water (BioLine, Taunton, MA). Two microliter of the serially diluted minigene solution was utilized for qPCR. A standard curve was generated using the relationship between the cycle numbers at threshold (Ct values) and the minigene copy numbers in serially diluted minigene solution.

### Statistical analysis

Unless otherwise stated statistical analysis were conducted using R 3.2.3 statistical software (https://cran.r-project.org/bin/windows/base/old/3.2.3/). OTUs accounting for < 0.005% of the total number of sequences were discarded before downstream analysis to reduce the problem of spurious OTUs [34]. The number of sequences varied markedly among individual mosquito midguts (mean ± SE = 6834.72 ± 460.75 per mosquito midgut; minimum = 0, maximum = 39,268). We rarefied the read depth to 1,036 reads per sample to standardize the sampling effort. Sixty nine samples that did not meet this criterion (i.e. had < 1,036 sequences) were excluded from subsequent analysis (Table 1). Alpha diversity metrics including Shannon diversity index, observed species, chao1, and evenness were generated in QIIME [35] and analysis of variance with Tukey adjustments was used to test whether there were any significant differences in these indices among mosquito species. Analysis of similarities (ANOSIM) using the “vegan” package in R was used to test whether microbial communities from samples of each mosquito species were more similar than those of different mosquito species [36]. The computed Bray-Curtis similarity matrix values were used for principal coordinate analysis (PCoA) to determine microbial community differences across mosquito species (“vegan” package in R). Hierarchical clusters based on Bray-Curtis dissimilarity measure were performed in PAST software to highlight the differences in mosquito samples based on the composition and abundance of their gut microbiota [37]. Similarity percentage (SIMPER) analysis was used to identify OTUs that were primarily responsible for observed differences between mosquito species (PAST version 3.14 software [37]).

## Results

### Bacterial species composition across mosquito species

MiSeq sequencing of the V3-V5 region of 16S rRNA gene amplicons from 264 mosquito samples generated a total of 1,804,366 sequences (Mean ± SE = 6834.72 ± 460.75 per mosquito midgut sample). After quality filtering and rarefying the reads to an even sampling depth of 1,036 sequences, a total of 202,020 sequences from 195 mosquito samples were retained. These sequences were clustered into 181 bacterial OTUs belonging to 11 phyla, 66 families and 111 genera. Only 16 of the 181 OTUs had an overall abundance equal to or greater than 1%. The majority of sequences were from *Proteobacteria* (81.1%) comprising of *Alphaproteobacteria* (47.4%), *Gammaproteobacteria* (29.2%), *Betaproteobacteria* (3.2%), *Epsilonproteobacteria* (1.1%), and *Deltaproteobacteria* (0.3%). Other observed phyla included, *Actinobacteria* (8.8%), *Firmicutes* (5.7%), *Bacteroidetes* (1.8%), *Acidobacteria* (0.8%), *Cyanobacteria* (0.6%), *Tenericutes* (0.5%), *Spirochaetes* (0.4%), *Planctomycetes* (0.3%), *Parcubacteria* (0.03%) and *Fusobacteria* (0.005%).

The most abundant OTUs were associated with the families *Acetobacteraceae* (25.7%), *Enterobacteriaceae* (20.6%), *Rickettsiaceae* (20.0%), *Propionibacteriaceae* (8.4%), and *Orbaceae* (4.2%) (S1 Fig). A*cetobacteraceae* occurred in high abundance among some individuals of all mosquito species except *An*. *crucians*. However, they were found in fewer individuals of *Ae*. *albopictus*, *An*. *punctipennis*, *An*. *quadrimaculatus*, *Cx*. *pipiens* and *Cx*. *restuans* compared to the remaining mosquito species. *Enterobacteriaceae* was more common among *Ae*. *triseriatus*, *Ae*. *trivittatus*, and *Ae*. *vexans* and also occurred in high abundance in the guts of a few individuals of the remaining mosquito species. *Rickettsiaceae* was more abundant and widespread in *Ae*. *albopictus* and *Cx*. *pipiens* and was also present in high abundance in a few samples of *An*. *crucians*, *An*. *punctipennis*, and *An*. *quadrimaculatus*. *Propionibacteriaceae* were mostly associated with *An*. *crucians* and *An*. *punctipennis* and occurred in high abundance in a few individuals of *Ae*. *triseriatus*, *Ae*. *vexans*, *An*. *quadrimaculatus*, *Cx*. *restuans*, and *Cs*. *inornata*. *Orbaceae* occurred in high abundance in a few individuals of *An*. *crucians*, *An*. *punctipennis*, *An*. *quadrimaculatus*, *Cs*. *inornata*, *Ps*. *ferox*, *Ae*. *japonicus* and *Ae*. *triseriatus*. Overall, only 1–3 major families of bacteria tended to dominate the guts of the 12 mosquito species (S1 Fig). It was also common for some individuals of a given mosquito species from the same study site and collection date to harbor different gut microbiota.

**Fig 2:**
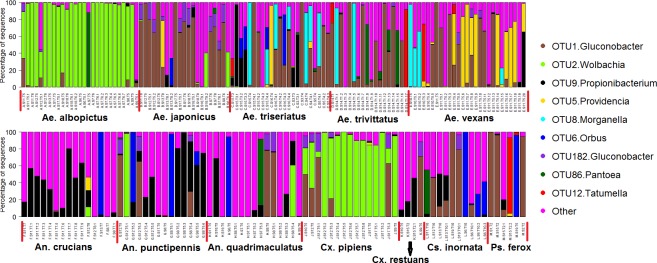
Mean relative abundances of bacterial OTUs associated with 12 species of mosquitoes at different sites and collection dates. OTUs with abundance of less than 1.5% were pooled together as “Other”. S = trap site, S1 = 504 S Lynn St, Champaign IL; S2 = 1509 Grandview Dr, Champaign IL; S3 = 408 W Maple St, Champaign IL; S4 = 1809 Grandview Dr, Champaign IL; S5 = 805 S. Prairie St, Champaign IL; S6 = 1116 Charles St, Champaign IL; S7 = 604 W Hill St, Champaign IL; S8 = 1605 Coronado Dr, Champaign IL; S9 = 1413 S Western Ave, Champaign IL; S10 = 909 S McKinley, Champaign IL; S11 = 1418 S Western Ave, Champaign IL; S12 = 409 W Hill St, Champaign IL; S13 = 705 W Kirby Ave, Champaign IL; S14 = 602 Nevada St, Urbana IL; S15 = 602 Vermont Ave, Urbana IL; S16 = 2402 E Elm St, Urbana IL; S17 = 804 W Main St, Urbana IL; S18 = 807 W Main St, Urbana IL; S 19 = 2010 Morrow Ct, Urbana IL. T = date of collection; T1 = July, 2, 2015; T2 = July, 7, 2015; T3 = July, 21, 2015; T4 = July, 28, 2015; T5 = August, 3, 2015; T6 = August, 11, 2015; T7 = August, 19, 2015; T8 = August, 28, 2015; T9 = September, 4, 2015; and T10 = October, 15, 2015.

The top 9 OTUs accounted for 69.2% of the total sequences and their relative abundance varied markedly between mosquito species (Fig 2). OTU 1 (*Gluconobacter*) accounted for 23.1% of the total sequences and was more abundant in all *Aedes* mosquito species (except *Ae*. *albopictus*) as well as *Cs*. *inornata* and *Ps*. *ferox*. This OTU also occurred in high abundance in a few samples of *Cx*. *pipiens*, *Cx*. *restuans*, *An*. *punctipennis*, and *An*. *quadrimaculatus*. OTU 2 (*Wolbachia*) was more prevalent and abundant in the guts of *Ae*. *albopictus and Cx*. *pipiens* and also occurred in three *Ae*. *japonicus* samples and one sample each of *An*. *crucians*, *An*. *punctipennis* and *An*. *quadrimaculatus*. OTU 9 (*Propionibacterium*) was mostly associated with *An*. *crucians* and *An*. *punctipennis* but it also occurred in higher abundance in a few samples of other mosquito species. OTU 8 (*Morganella*) was mostly associated with *Ae*. *triseriatus*, *Ae*. *trivittatus*, and *Ae*. *vexans* and OTU 5 (*Providencia*) was mostly associated with *Ae*. *vexans*. OTU 182 (*Gluconobacter*) was mostly associated with *Ae*. *japonicus* but was also present in high abundance in the guts of some individuals of other mosquito species. OTU 6 (*Orbus*), OTU 86 (*Pantoea*), and OTU 12 (*Tatumella*) occurred in high abundance in one or a few individuals of different mosquitoes (Fig 2). Some individuals of a given mosquito species also tended to differ in their microbial composition despite being collected from the same study sites and collection dates. The majority of mosquito samples were dominated by 1–2 OTUs.

**Fig 3:**
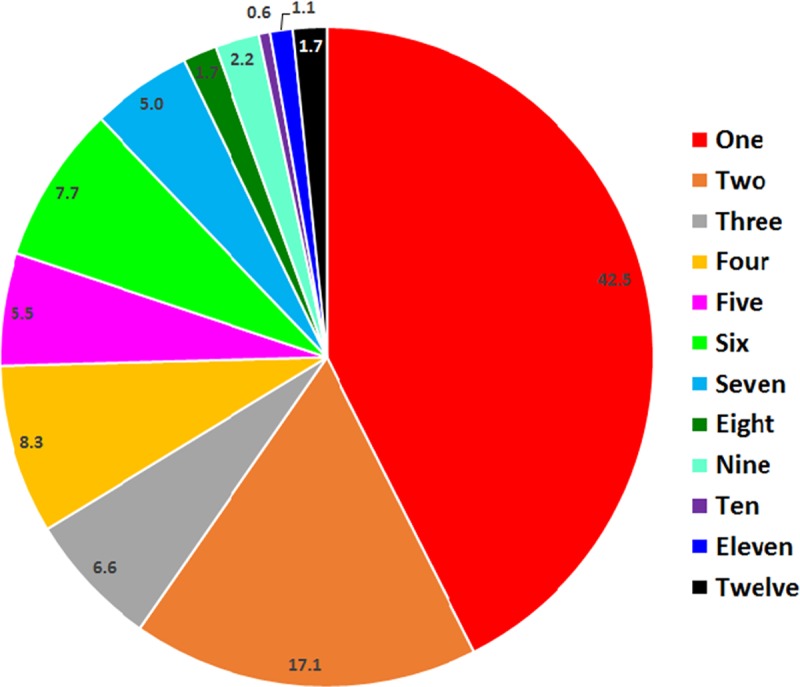
Number of OTUs that were unique to one mosquito species or shared between two or more mosquito species. n = 181.

Overall, 57.5% of bacterial OTUs were shared between at least two mosquito species (Fig 3). However, only three bacterial OTUs occurred in all 12 mosquito species. These were OTU 1 (*Gluconobacter*), OTU 9 (*Propionibacterium*), and OTU 31 (*Staphylococcus*).

### Diversity of mosquito microbiota

Shannon diversity indices revealed that on average, the gut microbiota of *Aedes albopictus* was the least diverse and significantly less even compared to gut microbiota of *An*. *crucians*, *An*. *quadrimaculatus*, *Ae*. *triseriatus*, *Ae*. *vexans*, *Ae*. *japonicus*, *Cx*. *restuans*, and *Cs*. *inornata* (Shannon: F = 6.4, df = 11, 179, *P* < 0.001; Evenness: F = 6.4, df = 11, 179, *P* < 0.001; Table 2). The gut microbiota of *An*. *crucians* was also significantly more diverse and more evenly distributed compared to that of *Ae*. *trivittatus*, *Cx*. *pipiens*, and *Ps*. *ferox* (Table 2). We also calculated Chao1 estimator based on OTUs abundance to determine the expected richness in each sample (Table 2). We were able to detect more than 93% ± 1.3% (mean ± SE) of the expected number of OTUs suggesting that most OTUs were recovered. On average, our results revealed that a mosquito midgut contains 5–10 bacterial OTUs (Table 2). The observed and predicted (Chao1) number of OTUs were significantly lower in *Ae*. *albopictus* compared to *Ae*. *vexans* (Observed OTUs: F = 3.2, 11, 179, *P* = 0.0005; Chao 1: F = 2.6, df = 11, 179, *P* = 0.005; Table 2). Significantly more bacterial OTUs were also observed in *An*. *crucians* and *Ae*. *triseriatus* guts compared to *Ae*. *albopictus* guts.

**Table 2:** Bacterial diversity and richness (mean ± SE) in the guts of 12 mosquito species.

Species	Shannon diversity	OTU evenness	Observed OTUS	Chao1
*Anopheles crucians*	1.87 ± 0.23	0.62 ± 0.06	8.00 ± 0.89	8.00 ± 0.89
*Anopheles punctipennis*	1.05 ± 0.15	0.39 ± 0.05	6.29 ± 0.57	7.04 ± 0.80
*Anopheles quadrimaculatus*	1.23 ± 0.20	0.45 ± 0.06	6.29 ± 0.53	6.82 ± 0.67
*Aedes albopictus*	0.36 ± 0.11	0.14 ± 0.03	4.50 ± 0.51	5.13 ± 0.67
*Aedes japonicus*	1.18 ± 0.17	0.43 ± 0.06	6.38 ± 0.47	7.14 ± 0.54
*Aedes triseriatus*	1.31 ± 0.15	0.45 ± 0.05	7.43 ± 0.68	8.41 ± 0.98
*Aedes vexans*	1.25 ± 0.11	0.41 ± 0.03	8.78 ± 0.88	9.82 ± 1.04
*Aedes trivittatus*	0.76 ± 0.12	0.31 ± 0.05	6.11 ± 0.50	6.58 ± 0.63
*Culex pipiens*	0.78 ± 0.15	0.29 ± 0.05	5.79 ± 0.59	6.18 ± 0.76
*Culex restuans*	1.61 ± 0.26	0.60 ± 0.09	6.50 ± 0.29	6.50 ± 0.29
*Culiseta inornata*	1.59 ± 0.33	0.54 ± 0.10	6.90 ± 0.99	7.15 ± 0.95
*Psorophora ferox*	0.65 ± 0.34	0.25 ± 0.10	5.83 ± 1.30	6.08 ± 1.37

### Variation in midgut bacterial communities across mosquito species

**Fig 4:**
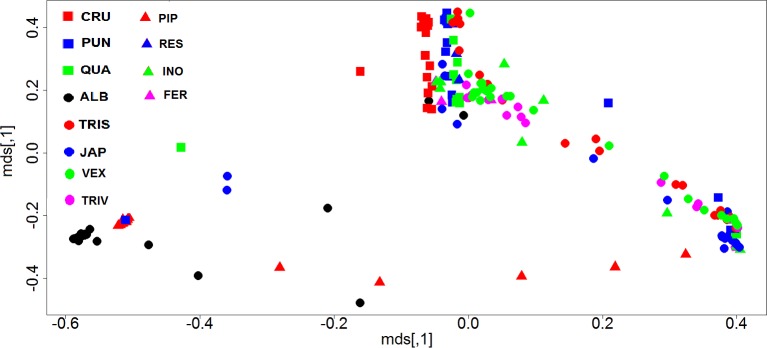
Principal coordinates analysis (PCoA) comparing the bacterial communities across 12 mosquito species. **PCoA was based on Bray-Curtis distance values computed for mosquito communities at the 97% OTU level.** CRU = *An*. *crucians*, PUN = *An*. *punctipennis*, QUA = *An*. *quadrimaculatus*, ALB = *Ae*. *albopictus*, JAP = *Ae*. *japonicus*, TRIS = *Ae*. *triseriatus*, VEX = *Ae*. *vexans*, TRIV = *Ae*. *trivittatus*, PIP = *Cx*. *pipiens*, RES = *Cx*. *restuans*, INO = *Cs*. *inornata*, FER = *Ps*. *ferox*.

The ANOSIM analysis based on Bray-Curtis distances revealed a significant difference in microbial communities among the 12 mosquito species (ANOSIM, *R* = 0.59, *P* = 0.001). To better visualize the results, a principal coordinates analysis (PCoA) plot was generated based on Bray-Curtis distances (Fig 4). Ordination based on this metric demonstrated a clear separation of *Ae*. *albopictus* and *Cx*. *pipiens* samples from the other mosquito species indicating that the microbial communities of the two mosquito species differed from those of the other mosquito species (Fig 4). Cluster analysis based on Bray-Curtis distances confirmed that the majority of *Ae*. *albopictus* and *Cx*. *pipiens* samples tended to cluster together and that it was common for individuals of different mosquito species from different sites and collection dates to harbor similar gut microbiota (S2 Fig).

The SIMPER analysis was used to identify the bacterial OTUs primarily responsible for the observed separation of gut communities between mosquito species, using the relative abundances of bacterial OTUs (S1 Table; S3 Fig). Twelve OTUs accounted for 69.8% of observed differences between mosquito species with OTU 1 (19%), OTU 2 (18%) and OTU 9 (8%) accounting for the largest variation (S1 Table). OTU 1 (*Gluconobacter*), was found in all mosquito species but was more abundant in *Ae*. *japonicus*, *Ps*. *ferox*, *Ae*. *trivittatus*, *Ae*. *triseriatus*, and *Cs*. *inornata* (S3 Fig). OTU 2 (*Wolbachia*) was mainly associated with *Ae*. *albopictus* and *Cx*. *pipiens* and OTU 9 (*Propionibacterium*) was mainly associated *Cx*. *restuans*, *Ae*. *triseriatus* and the three *Anopheles* species (*An*. *crucians*, *An*. *quadrimaculatus*, *An*. *punctipennis*, S3 Fig).

### *Wolbachia* prevalence and wsp gene copy numbers

Real-time qPCR results confirmed the presence of *Wolbachia* in all three *Ae*. *japonicus* samples, 25 of 27 *Ae*. *albopictus* samples, 12 of 15 *Cx*. *pipiens* samples, and the 1 *An*. *punctipennis* sample (S4 Fig). None of the other mosquito species had *Wolbachia*. *Wolbachia* wsp gene copy numbers ranged from 0 to 10151 and were relatively higher in *Ae*. *albopictus* compared to the other mosquito species (S4 Fig).

## Discussion

In this study we characterized and compared the midgut bacterial communities of 12 mosquito species encompassing four mosquito genera, many of them important vectors of medical, veterinary and wildlife significance. Overall, we found a low diversity of gut microbiota that was characterized by large individual variability and the dominance of one or two bacterial OTUs. Analysis of microbial composition revealed that the bacterial community in mosquito midguts was dominated by a few phyla with only three phyla (*Proteobacteria* (81.1%), *Actinobacteria* (8.8%) and *Firmicutes* (5.7%) accounting for 97% of the total sequences. These bacterial phyla are commonly reported in the guts of mosquitoes and other insects [22, 24, 25, 38, 39]. The Phylum *Proteobacteria* is highly diverse and contains a wide variety of species that are adapted to a wide range of environments; thus it is no surprise that its dominance in mosquito midguts is well established [22, 24, 25, 40, 41].

Individual variability in gut microbiota was not only restricted to mosquito samples collected from different sites and different dates but was also common among individual mosquitoes collected at the same sites and collection dates. Similar individual variability in gut microbiota and the dominance of a few bacterial taxa in mosquito guts has been reported before [22]. These variations may result from individual variations in external and internal factors such as the gut physiological conditions, larval and adult diet, infection with parasites and pathogens, host aging [24, 26, 27, 38, 42], and host genetic background [43]. Our experimental design cannot decipher the contribution of these factors to the observed pattern of gut microbiota since adult mosquito samples were collected using the CDC light traps and we had no prior knowledge of the factors these mosquitoes were exposed to before collection. Individual variation in gut microbiota may be epidemiologically relevant since some bacterial species are known to enhance [13, 44, 45] or reduce mosquito susceptibility to *Plasmodium* parasites and dengue viruses [14, 46, 47]. Thus it is possible that differences in gut microbiota observed in this study may be one of the primary factors contributing to individual variation in vector competence that is commonly observed in nature. Future studies targeting the role of specific members of this bacterial community on vector competence and other aspects of mosquito biology may provide important insights into their epidemiological significance.

*Ae*. *albopictus* and *Cx*. *pipiens* harbored distinct bacterial communities that was primarily dominated by OTU 2 (*Wolbachia*). We also found *Wolbachia* sequences in three samples of *Ae*. *japonicus* and one sample of *An*. *crucians*, *An*. *quadrimaculatus*, and *An*. *punctipennis*. Real-time qPCR results confirmed the widespread occurrence of *Wolbachia* in *Ae*. *albopictus* and *Cx*. *pipiens* samples as well as its presence in the 1 and 3 *An*. *punctipennis* and *Ae*. *japonicus* samples that had *Wolbachia* sequences, respectively. We processed only intact mosquitoes and sterilized their surfaces before dissecting their midguts to minimize the potential for contamination. This process is expected to remove bacteria from the body surface but it is still possible these mosquitoes were contaminated with *Wolbachia* from damaged *Ae*. *albopictus* and *Cx*. *pipiens* samples either in the traps or during sorting and sample identification. However, the dominance of *Wolbachia* sequences in one of *An*. *punctipennis* samples and three *Ae*. *japonicus* samples is unlikely due to cross contamination and may imply that a few individuals of *Ae*. *japonicus* and *An*. *punctipennis* may harbor *Wolbachia* endosymbionts. *Wolbachia* are a genus of maternally-inherited bacterial endosymbionts that are estimated to occur in approximately 65% of insect species [48]. This bacterium acts as a reproductive parasite in arthropods; it induces male killing, feminization, and cytoplasmic incompatibility which facilitate its spread throughout the arthropod population [49]. Both *Ae*. *albopictus* and *Cx*. *pipiens* are known to harbor *Wolbachia* endosymbionts [23, 38, 50–52] and our study suggest the need for detailed investigations of *Wolbachia* infection to ascertain that its absence in other mosquito species as reported in the past is not due to lack of adequate sampling effort. The mechanism underlying the high *Wolbachia* infection and low diversity of midgut bacteria in *Ae*. *albopictus* is unclear but could be due to methodological bias where the rarefaction depth of 1,036 employed in this study may not have been sufficient to detect low abundance OTUs or due to *Wolbachia* interacting negatively with other bacterial species. Additional studies are needed to develop a better understanding of how *Wolbachia* interacts with other microbiota. *Wolbachia* has been shown to inhibit transmission of mosquito-borne pathogens [53–55] and is currently under investigation for potential application in biological control of mosquitoes and associated pathogens [56–58]. Unfortunately, *Wolbachia* can also enhance transmission of other pathogens such as malaria and West Nile Virus [44, 45, 59]. These effects are dependent on *Wolbachia* strain and the mosquito-borne pathogen under investigation as it is possible for some *Wolbachia* strains to inhibit transmission of some pathogens while enhancing transmission of others [60, 61]. These findings reinforce the need to understand the potential impact of *Wolbachia* on different mosquito-borne pathogens before large scale application of *Wolbachia*-based disease control strategies.

SIMPER analyses indicated that OTU 1 (*Gluconobacter*), OTU 2 (*Wolbachia*), and OTU 9 (*Propionibacterium*) contributed most to the average dissimilarity between mosquito species. OTU 1 (*Gluconobacter*) was found in all mosquito species but was strongly associated with *Ae*. *japonicus*, *Ae*. *triseriatus*, *Ae*. *vexans*, *Ae*. *trivittatus*, *Cs*. *inornata*, and *Ps*. *ferox*. *Gluconobacter* are acetic acid bacteria that are adapted to various sugar- and ethanol-rich environments [62]. These bacteria have been found in association with insects that rely on sugar-based diets including mosquitoes [63, 64]. As an example, the genus *Asaia* (a member of *Acetobacteraceae*), are frequently found in the nectar of flowers e.g. [65–67] and have been shown to establish symbiotic associations with mosquitoes [63, 64, 68, 69]. *Propionibacterium* was mostly associated with *Anopheles* mosquitoes and *Cx*. *restuans*. *Propionibacterium* is a common bacteria of human skin and other animals [70–72] and has also been isolated in mosquitoes [73]. These bacteria may have been acquired from vertebrate hosts during a blood meal [73]. Another notable OTU accounting for observed differences was OTU 5 (*Providencia*) which was strongly associated with *Ae*. *vexans*. This bacterium is a common gastrointestinal pathogen of humans and animals and also occurs in human and animal wastes [74]. It may have been acquired through contact with blood meal hosts or during larval development. Further studies are needed to investigate the potential role of these bacteria on mosquito biology including susceptibility to pathogens.

In general, there were small differences in bacterial diversity and evenness between most species of mosquitoes. However, the bacterial communities of *Ae*. *albopictus* were significantly less diverse and less evenly distributed compared to those of *An*. *crucians*, *An*. *quadrimaculatus*, *Ae*. *japonicus*, *Ae*. *triseriatus*, *Ae*. *vexans*, *Cx*. *restuans*, or *Cs*. *inornata*. Similar bacterial diversity and evenness between mosquito species across the four mosquito genera suggest that the mosquito midgut likely plays an active role in regulating the colonization and assembly of bacterial communities. Lower microbial diversity in *Ae*. *albopictus* relative to the seven mosquito species may be due to inability of some bacterial taxa to proliferate in the guts of *Ae*. *albopictus* either due to species differences in gut physiological conditions [75] and/or modulation of microbial communities by the mosquito innate immune system [12]. The physical presence of some bacterial taxa or other microbes (e.g. fungi) also may render the mosquito midgut uninhabitable to other bacterial taxa due to interspecific competition for resources and/or production of toxins and inhibitory factors. Differences in food sources also may partly account for the observed differences because although all mosquito species tend to feed on microbes as larvae and blood and nectar as adults, different mosquito species portray marked variations in their preferred larval habitats and sugar and blood meal hosts which may pre-expose them to different microorganisms. In addition, sugar feeding and blood feeding can reduce the diversity of gut bacteria in mosquitoes [24]. Although we purposefully selected individuals that were not engorged with blood for microbiome analysis, we could not establish whether our mosquito samples had prior access to a blood meal or a sugar meal. It is possible that the majority of *Ae*. *albopictus* that were analyzed in this study had acquired a blood meal and/or a sugar meal leading to major reductions in bacterial diversity.

In summary, our study has characterized the midgut bacterial communities of 12 of the most common mosquito species in the United States, expanding current knowledge on mosquito species whose gut microbes have been studied. We found significant differences in gut microbial composition between some mosquito species and documented marked variation in gut microbiota between individuals of the same mosquito species. The 12 mosquito species included the known vectors of arboviruses of global public health significance such as dengue, chikungunya, Zika, West Nile virus, and La Crosse virus encephalitis. Given the well-documented ability of midgut microbiota to influence vector susceptibility to pathogens [12, 14–16, 25, 46], our results provide critical knowledge that can inspire further studies to determine which of the identified microbial communities could be exploited for disease control.

## Supporting information

S1 Fig Mean relative abundances of bacterial families associated with 12 species of mosquitoes at different sites and collection dates. Families with abundance of less than 1.2% were pooled together as “Other”. S = trap site, S1 = 504 S Lynn St, Champaign IL; S2 = 1509 Grandview Dr, Champaign IL; S3 = 408 W Maple St, Champaign IL; S4 = 1809 Grandview Dr, Champaign IL; S5 = 805 S. Prairie St, Champaign IL; S6 = 1116 Charles St, Champaign IL; S7 = 604 W Hill St, Champaign IL; S8 = 1605 Coronado Dr, Champaign IL; S9 = 1413 S Western Ave, Champaign IL; S10 = 909 S McKinley, Champaign IL; S11 = 1418 S Western Ave, Champaign IL; S12 = 409 W Hill St, Champaign IL; S13 = 705 W Kirby Ave, Champaign IL; S14 = 602 Nevada St, Urbana IL; S15 = 602 Vermont Ave, Urbana IL; S16 = 2402 E Elm St, Urbana IL; S17 = 804 W Main St, Urbana IL; S18 = 807 W Main St, Urbana IL; S 19 = 2010 Morrow Ct, Urbana IL. T = date of collection; T1 = July, 2, 2015; T2 = July, 7, 2015; T3 = July, 21, 2015; T4 = July, 28, 2015; T5 = August, 3, 2015; T6 = August, 11, 2015; T7 = August, 19, 2015; T8 = August, 28, 2015; T9 = September, 4, 2015; and T10 = October, 15, 2015.
(TIFF)

S2 Fig Clustering based on taxon composition and abundance of gut microbiota of all mosquito samples. CRU = *An*. *crucians*, PUN = *An*. *punctipennis*, QUA = *An*. *quadrimaculatus*, ALB = *Ae*. *albopictus*, JAP = *Ae*. *japonicus*, TRIS = *Ae*. *triseriatus*, VEX = *Ae*. *vexans*, TRIV = *Ae*. *trivittatus*, PIP = *Cx*. *pipiens*, RES = *Cx*. *restuans*, INO = *Cs*. *inornata*, FER = *Ps*. *ferox*. S = trap site, S1 = 504 S Lynn St, Champaign IL; S2 = 1509 Grandview Dr, Champaign IL; S3 = 408 W Maple St, Champaign IL; S4 = 1809 Grandview Dr, Champaign IL; S5 = 805 S. Prairie St, Champaign IL; S6 = 1116 Charles St, Champaign IL; S7 = 604 W Hill St, Champaign IL; S8 = 1605 Coronado Dr, Champaign IL; S9 = 1413 S Western Ave, Champaign IL; S10 = 909 S McKinley, Champaign IL; S11 = 1418 S Western Ave, Champaign IL; S12 = 409 W Hill St, Champaign IL; S13 = 705 W Kirby Ave, Champaign IL; S14 = 602 Nevada St, Urbana IL; S15 = 602 Vermont Ave, Urbana IL; S16 = 2402 E Elm St, Urbana IL; S17 = 804 W Main St, Urbana IL; S18 = 807 W Main St, Urbana IL; S 19 = 2010 Morrow Ct, Urbana IL. T = date of collection; T1 = July, 2, 2015; T2 = July, 7, 2015; T3 = July, 21, 2015; T4 = July, 28, 2015; T5 = August, 3, 2015; T6 = August, 11, 2015; T7 = August, 19, 2015; T8 = August, 28, 2015; T9 = September, 4, 2015; and T10 = October, 15, 2015.
(TIF)

S3 Fig Principal OTUs responsible for observed differences in bacterial community structure between mosquito species. CRU = *An*. *crucians*, PUN = *An*. *punctipennis*, QUA = *An*. *quadrimaculatus*, ALB = *Ae*. *albopictus*, JAP = *Ae*. *japonicus*, TRIS = *Ae*. *triseriatus*, VEX = *Ae*. *vexans*, TRIV = *Ae*. *trivittatus*, PIP = *Cx*. *pipiens*, RES = *Cx*. *restuans*, INO = *Cs*. *inornata*, FER = *Ps*. *ferox*. Number at the end of the genus name is the OTU number. Values in the heatmap cells represent the relative abundance of respective OTUs in different mosquito species.
(TIF)

S4 Fig qPCR results of *Wolbachia* wsp copy numbers in *Aedes albopictus*, *Ae*. *japonicus*, *Anopheles crucians*, *An*. *punctipennis*, *An*. *quadrimaculatus*, and *Culex pipiens*. S = trap site, S1 = 504 S Lynn St, Champaign IL; S2 = 1509 Grandview Dr, Champaign IL; S3 = 408 W Maple St, Champaign IL; S4 = 1809 Grandview Dr, Champaign IL; S5 = 805 S. Prairie St, Champaign IL; S6 = 1116 Charles St, Champaign IL; S7 = 604 W Hill St, Champaign IL; S8 = 1605 Coronado Dr, Champaign IL; S9 = 1413 S Western Ave, Champaign IL; S10 = 909 S McKinley, Champaign IL; S11 = 1418 S Western Ave, Champaign IL; S12 = 409 W Hill St, Champaign IL; S13 = 705 W Kirby Ave, Champaign IL; S14 = 602 Nevada St, Urbana IL; S15 = 602 Vermont Ave, Urbana IL; S16 = 2402 E Elm St, Urbana IL; S17 = 804 W Main St, Urbana IL; S18 = 807 W Main St, Urbana IL; S 19 = 2010 Morrow Ct, Urbana IL. T = date of collection; T1 = July, 2, 2015; T2 = July, 7, 2015; T3 = July, 21, 2015; T4 = July, 28, 2015; T5 = August, 3, 2015; T6 = August, 11, 2015; T7 = August, 19, 2015; T8 = August, 28, 2015; T9 = September, 4, 2015; and T10 = October, 15, 2015.
(TIF)

S1 Table SIMPER analysis showing the major OTUs contributing to group differences. (DOCX)
