# Supplementary figures and images for: Comparative analysis of gut microbiota of mosquito communities in central Illinois
Source: PLoS Negl Trop Dis. 2017 Feb 28;11(2):e0005377. doi: 10.1371/journal.pntd.0005377 (PMC5345876; doi:10.1371/journal.pntd.0005377)

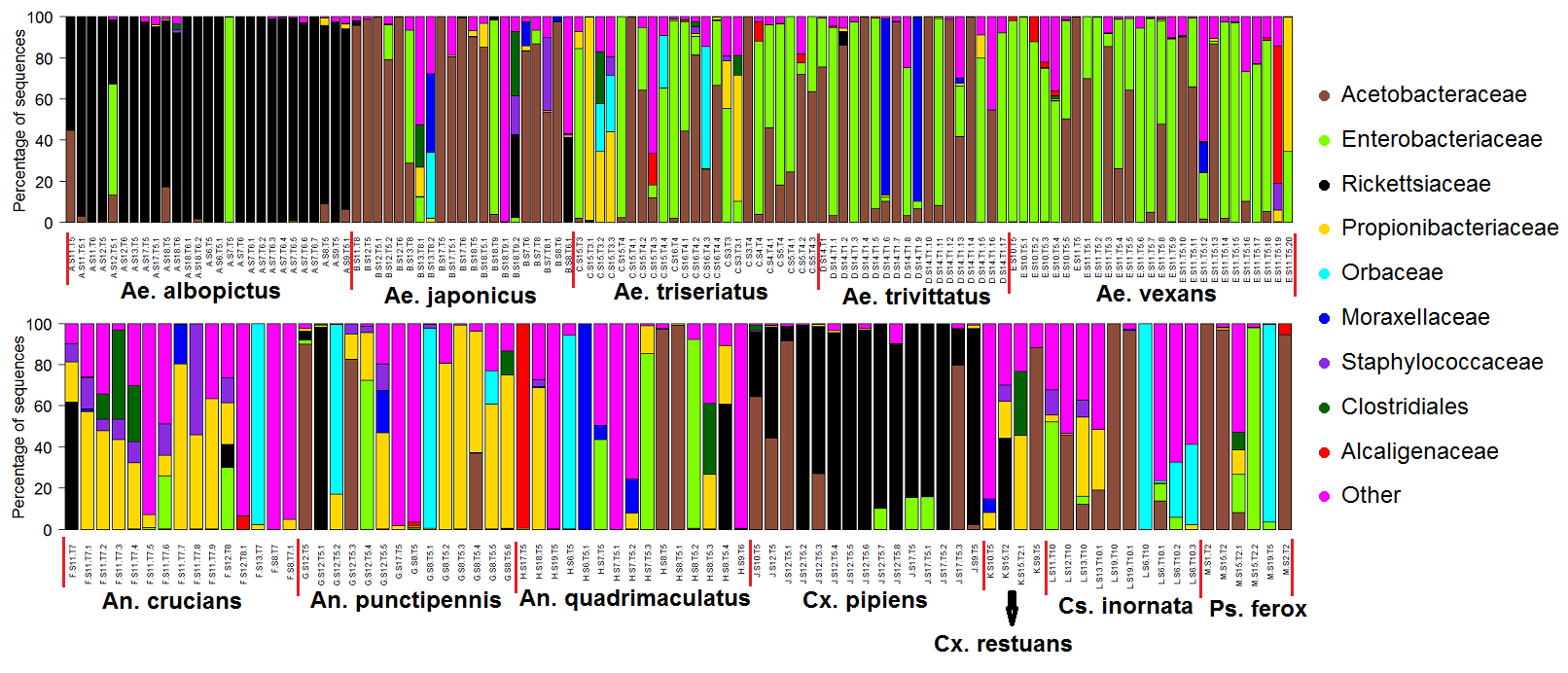

Supplement: S1 Fig — Families with abundance of less than 1.2% were pooled together as “Other”. S = trap site, S1 = 504 S Lynn St, Champaign IL; S2 = 1509 Grandview Dr, Champaign IL; S3 = 408 W Maple St, Champaign IL; S4 = 1809 Grandview Dr, Champaign IL; S5 = 805 S. Prairie St, Champaign IL; S6 = 1116 Charles St, Champaign IL; S7 = 604 W Hill St, Champaign IL; S8 = 1605 Coronado Dr, Champaign IL; S9 = 1413 S Western Ave, Champaign IL; S10 = 909 S McKinley, Champaign IL; S11 = 1418 S Western Ave, Champaign IL; S12 = 409 W Hill St, Champaign IL; S13 = 705 W Kirby Ave, Champaign IL; S14 = 602 Nevada St, Urbana IL; S15 = 602 Vermont Ave, Urbana IL; S16 = 2402 E Elm St, Urbana IL; S17 = 804 W Main St, Urbana IL; S18 = 807 W Main St, Urbana IL; S 19 = 2010 Morrow Ct, Urbana IL. T = date of collection; T1 = July, 2, 2015; T2 = July, 7, 2015; T3 = July, 21, 2015; T4 = July, 28, 2015; T5 = August, 3, 2015; T6 = August, 11, 2015; T7 = August, 19, 2015; T8 = August, 28, 2015; T9 = September, 4, 2015; and T10 = October, 15, 2015. (TIFF) [file pntd.0005377.s001.tiff]

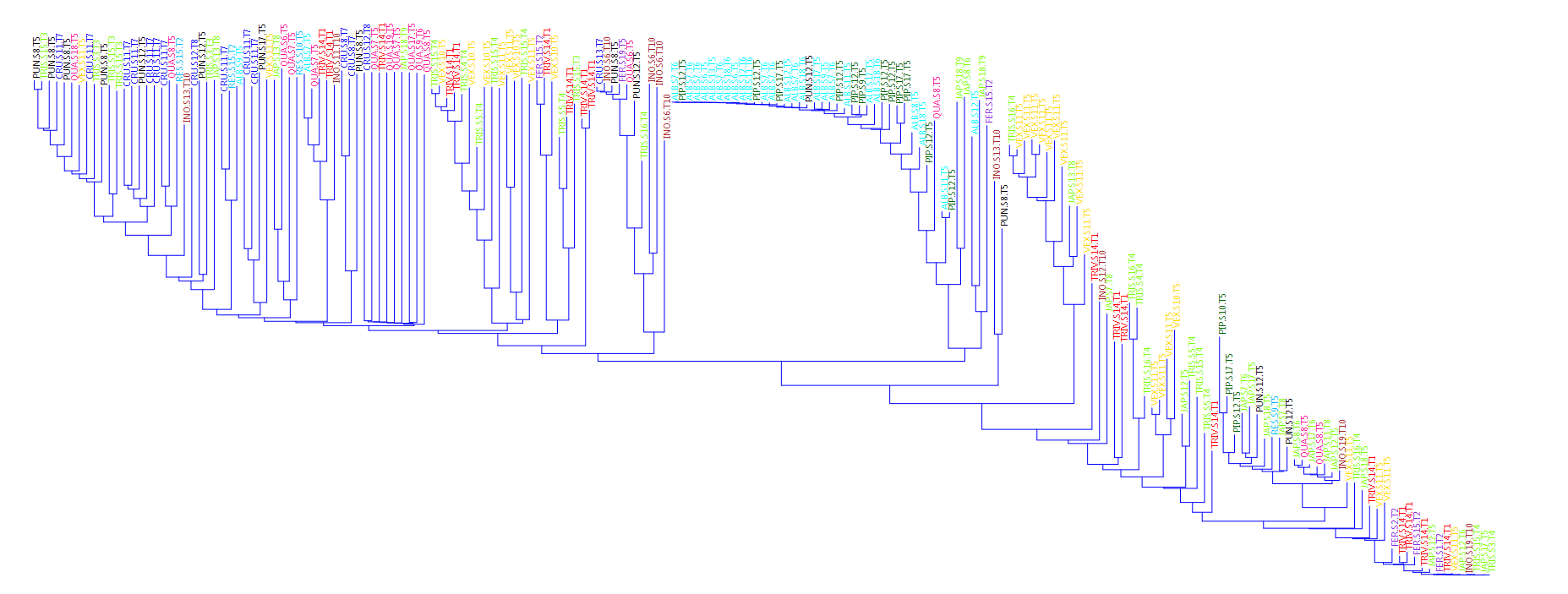

Supplement: S2 Fig — CRU = An. crucians, PUN = An. punctipennis, QUA = An. quadrimaculatus, ALB = Ae. albopictus, JAP = Ae. japonicus, TRIS = Ae. triseriatus, VEX = Ae. vexans, TRIV = Ae. trivittatus, PIP = Cx. pipiens, RES = Cx. restuans, INO = Cs. inornata, FER = Ps. ferox. S = trap site, S1 = 504 S Lynn St, Champaign IL; S2 = 1509 Grandview Dr, Champaign IL; S3 = 408 W Maple St, Champaign IL; S4 = 1809 Grandview Dr, Champaign IL; S5 = 805 S. Prairie St, Champaign IL; S6 = 1116 Charles St, Champaign IL; S7 = 604 W Hill St, Champaign IL; S8 = 1605 Coronado Dr, Champaign IL; S9 = 1413 S Western Ave, Champaign IL; S10 = 909 S McKinley, Champaign IL; S11 = 1418 S Western Ave, Champaign IL; S12 = 409 W Hill St, Champaign IL; S13 = 705 W Kirby Ave, Champaign IL; S14 = 602 Nevada St, Urbana IL; S15 = 602 Vermont Ave, Urbana IL; S16 = 2402 E Elm St, Urbana IL; S17 = 804 W Main St, Urbana IL; S18 = 807 W Main St, Urbana IL; S 19 = 2010 Morrow Ct, Urbana IL. T = date of collection; T1 = July, 2, 2015; T2 = July, 7, 2015; T3 = July, 21, 2015; T4 = July, 28, 2015; T5 = August, 3, 2015; T6 = August, 11, 2015; T7 = August, 19, 2015; T8 = August, 28, 2015; T9 = September, 4, 2015; and T10 = October, 15, 2015. (TIF) [file pntd.0005377.s002.tif]

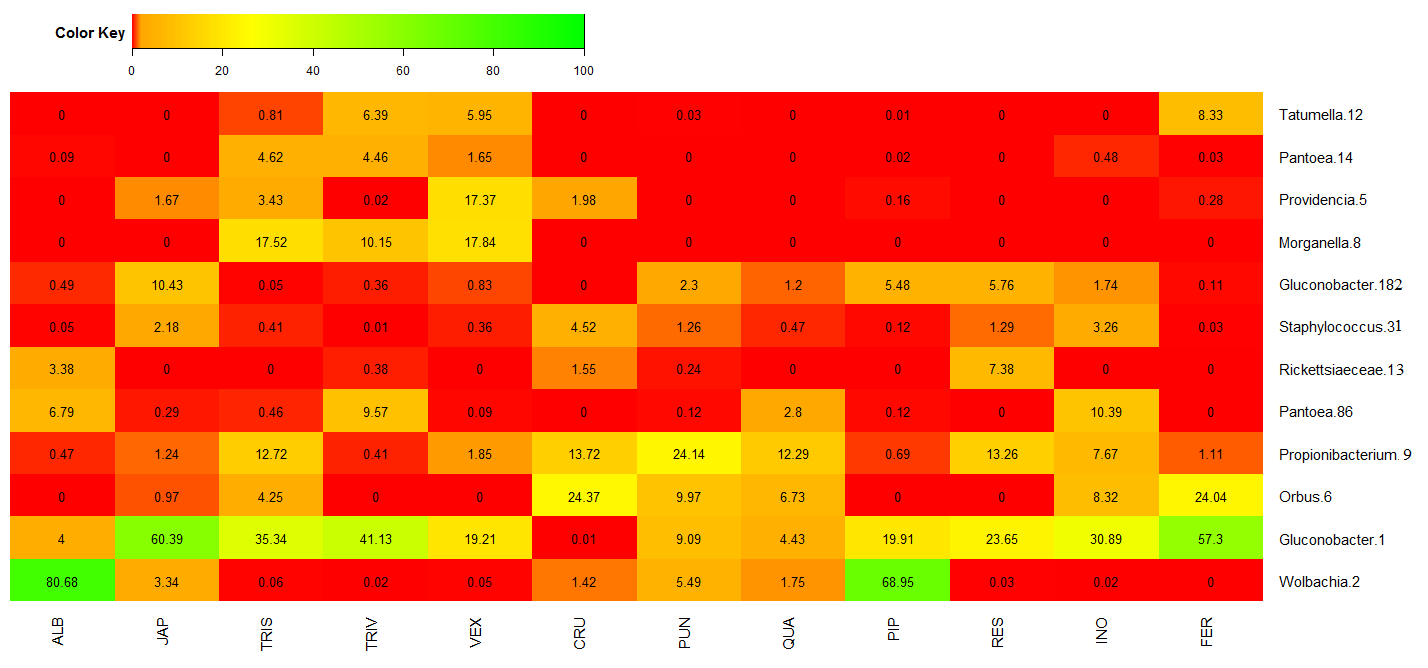

Supplement: S3 Fig — CRU = An. crucians, PUN = An. punctipennis, QUA = An. quadrimaculatus, ALB = Ae. albopictus, JAP = Ae. japonicus, TRIS = Ae. triseriatus, VEX = Ae. vexans, TRIV = Ae. trivittatus, PIP = Cx. pipiens, RES = Cx. restuans, INO = Cs. inornata, FER = Ps. ferox. Number at the end of the genus name is the OTU number. Values in the heatmap cells represent the relative abundance of respective OTUs in different mosquito species. (TIF) [file pntd.0005377.s003.tif]

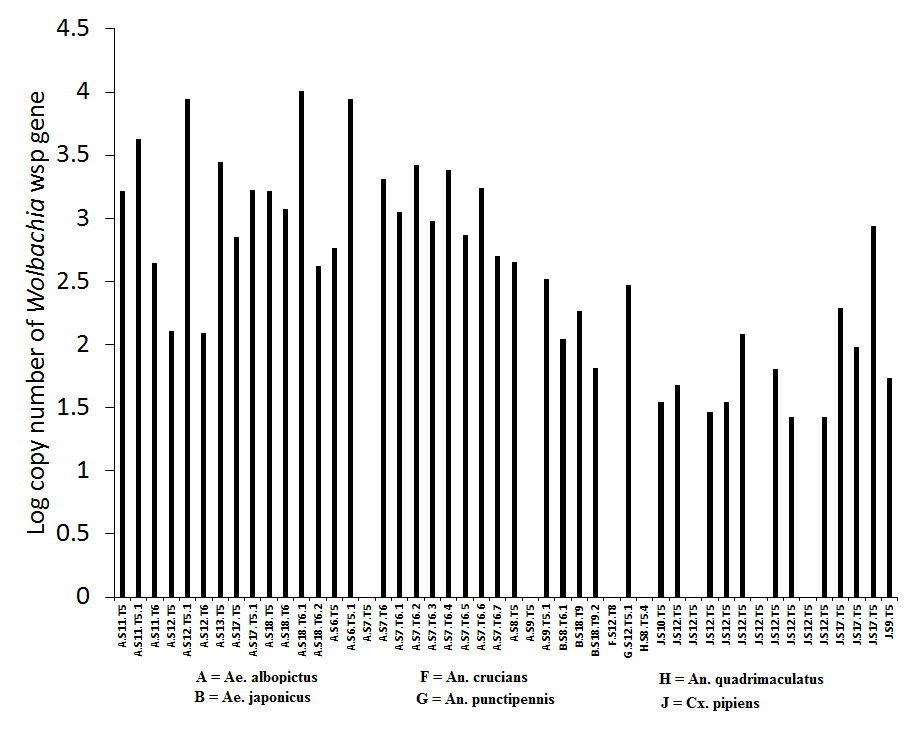

Supplement: S4 Fig — S = trap site, S1 = 504 S Lynn St, Champaign IL; S2 = 1509 Grandview Dr, Champaign IL; S3 = 408 W Maple St, Champaign IL; S4 = 1809 Grandview Dr, Champaign IL; S5 = 805 S. Prairie St, Champaign IL; S6 = 1116 Charles St, Champaign IL; S7 = 604 W Hill St, Champaign IL; S8 = 1605 Coronado Dr, Champaign IL; S9 = 1413 S Western Ave, Champaign IL; S10 = 909 S McKinley, Champaign IL; S11 = 1418 S Western Ave, Champaign IL; S12 = 409 W Hill St, Champaign IL; S13 = 705 W Kirby Ave, Champaign IL; S14 = 602 Nevada St, Urbana IL; S15 = 602 Vermont Ave, Urbana IL; S16 = 2402 E Elm St, Urbana IL; S17 = 804 W Main St, Urbana IL; S18 = 807 W Main St, Urbana IL; S 19 = 2010 Morrow Ct, Urbana IL. T = date of collection; T1 = July, 2, 2015; T2 = July, 7, 2015; T3 = July, 21, 2015; T4 = July, 28, 2015; T5 = August, 3, 2015; T6 = August, 11, 2015; T7 = August, 19, 2015; T8 = August, 28, 2015; T9 = September, 4, 2015; and T10 = October, 15, 2015. (TIF) [file pntd.0005377.s004.tif]
