# Supplementary material for: Comparative analysis of gut microbiota of mosquito communities in central Illinois
Source: PLoS Negl Trop Dis. 2017 Feb 28;11(2):e0005377. doi: 10.1371/journal.pntd.0005377 (PMC5345876; doi:10.1371/journal.pntd.0005377)
Supplement: S1 Table — (DOCX) [file pntd.0005377.s005.docx]

**S1 Table: SIMPER analysis showing the major OTUs contributing to group differences**

| OTUID | Class | Family | Genus | Average dissimilarity | Contribution (%) |
| --- | --- | --- | --- | --- | --- |
| 1 | Alphaproteobacteria | Acetobacteraceae | *Gluconobacter* | 17.01 | 19 |
| 2 | Alphaproteobacteria | Anaplasmataceae | *Wolbachia* | 16.49 | 18.41 |
| 9 | Actinobacteria | Propionibacteriaceae | *Propionibacterium* | 7.356 | 8.212 |
| 5 | Gammaproteobacteria | Enterobacteriaceae | *Providencia* | 4.25 | 4.745 |
| 6 | Gammaproteobacteria | Orbaceae | *Orbus* | 4.131 | 4.612 |
| 8 | Gammaproteobacteria | Enterobacteriaceae | *Morganella* | 3.974 | 4.437 |
| 86 | Gammaproteobacteria | Enterobacteriaceae | *Pantoea* | 2.078 | 2.32 |
| 182 | Alphaproteobacteria | Acetobacteraceae | *Gluconobacter* | 1.976 | 2.206 |
| 12 | Gammaproteobacteria | Enterobacteriaceae | *Tatumella* | 1.59 | 1.775 |
| 31 | Firmicutes | Staphylococcaceae | *Staphylococcus* | 1.333 | 1.488 |
| 14 | Gammaproteobacteria | Enterobacteriaceae | *Pantoea* | 1.241 | 1.386 |
| 13 | Alphaproteobacteria | Rickettsiaceae | *Rickettsiaceae* | 1.049 | 1.172 |
